# Environmental and health effects of the Barcelona superblocks

**DOI:** 10.1186/s12889-025-21835-z

**Published:** 2025-02-17

**Authors:** Katherine Pérez, Laia Palència, Maria José López, Brenda Biaani León-Gómez, Anna Puig-Ribera, Anna Gómez-Gutiérrez, Mark Nieuwenhuijsen, Glòria Carrasco-Turigas, Carme Borrell

**Affiliations:** 1 Agència de Salut Pública de Barcelona (ASPB), Plaça Lesseps 1, 08023 Barcelona, Spain; 2 CIBER Epidemiología y Salud Pública (CIBERESP), Madrid, Spain; 3 Institut de Recerca Sant Pau (IR SANT PAU), Barcelona, Spain; 4 Universitat Pompeu Fabra, Barcelona, Spain; 5 Unitat de Suport a La Recerca (USR) Metropolitana Nord, Mataró, Spain; 6 Institut Universitari d’Investigació en Atenció Primària Jordi Gol (IDIAP Jordi Gol), Barcelona, Spain; 7 Sport and Physical Activity Research Group, Institute for Research and Innovation in Life Sciences and Health in Central Catalonia, University of Vic-Central University of Catalonia, Vic, Spain; 8 ISGlobal, Barcelona, Spain

**Keywords:** Program evaluation, Quality of life, City planning, Health determinants, Health in all policies

## Abstract

**Background:**

The superblocks model of Barcelona (Spain) seeks to reorganize the city based on reversing the distribution of public space between vehicles and people by prioritizing citizens, thus improving their environmental conditions and quality of life. The objective of this paper was to describe the effects on environmental, health and quality of life of the first three superblocks implemented, discuss the lessons learned, and provide recommendations for the future.

**Methods:**

The evaluation included different approaches depending on the superblock analyzed: A pre-post-intervention health survey, environmental measures of air quality, the Microscale Audit of Urban Landscapes for Pedestrians (MAPS), an observational study on target areas of physical activity (SOPARC), ethnographic guerrilla studies, and focus groups.

**Results:**

Residents and workers in all three of the evaluated superblocks reported a perceived improvement in well-being, tranquillity and quality of sleep, some reduction in noise and pollution and an increase in social interaction. The built environment of the superblocks clearly influenced walkability, and the lower car traffic volume improved air quality measures. In one of the superblocks, the same intervention calmed traffic in one of the areas but not in another.

**Conclusions:**

Although on a small scale, this evaluation of the environmental, and health effects of the superblocks provides support for expanding the model to other areas of the city.

**Supplementary Information:**

The online version contains supplementary material available at 10.1186/s12889-025-21835-z.

## Introduction

Urban areas face multiple challenges in terms of urban planning, environmental issues, and public health. These challenges include high levels of noise and air pollution, the urban heat island effect, limited green and living spaces, and the need to improve mental health, well-being and other health outcomes, such as reducing traffic injuries [1]. Moreover, cities contribute to climate change through activities that release greenhouse gas emissions, mainly caused by urban planning, such as transportation and buildings [2]. Consequently, these areas are also part of the solution to climate change [3]. At the same time, urban areas are particularly susceptible to the health impacts of climate change. Indeed, in Mediterranean cities, these impacts place additional pressure on already-stressed ecosystems and vulnerable economies and societies, increasing health inequalities[4]. Therefore, urban planning is crucial to identify solutions to these problems and mitigate the effects of climate change. In Spain urban planning is a responsibility of the government of the city.

The city of Barcelona faces major environmental and health challenges that could be mitigated by improved urban planning [5]. In recent years, the Barcelona City Council has begun to implement the superblocks model in several neighborhoods of the city, as part of the government’s ‘Let's fill the streets with life’ initiative [6]. It aims to reorganize the city by reversing the distribution of public space between vehicles and people and prioritizing citizens to enhance environmental conditions and quality of life [6]. This program aims to improve the liveability of public spaces, promote sustainable mobility, enhance urban green spaces, and encourage citizen participation and co-responsibility [6]. The initiative actively seeks citizen participation and the involvement and co-responsibility of the existing social fabric in each area during the implementation process. The types of intervention can vary according to the extent to which actions are divided into basic (changes to street functions), tactical (low-budget, temporary and reversible innovations that serve to test models and change the uses of the streets) and structural (actions requiring greater consensus, long-term stability and that may involve larger budgets). The interventions focus on traffic calming around several city blocks and giving priority to pedestrians and cyclists, thereby opening up a number of streets to citizens.

A conceptual framework for evaluating the health impacts of superblocks was developed by Mehdipanah et al. [7]. This framework shows how urban governance, through the implementation of superblocks, aims to affect public space, the various types of mobility, the amount of green space, and community participation. This intervention is expected to affect both the neighbourhood (decreased air and noise pollution, increased road safety and walkability, etc.) and individuals (increased active transportation and social interaction, etc.). However, the intervention could also increase the cost of living and housing and have undesired effects such as the possible expulsion of residents from the neighbourhood (gentrification). All these factors will have effects on health and, if these effects vary according to the different axes of inequality, will have also effects on social inequalities in health.

The first three superblocks implemented in Barcelona were in the neighbourhoods of Poblenou, Sant Antoni, and Horta. Each of these neighbourhoods has a different population, infrastructure and intervention characteristics [6]. Each superblock reorganizes traffic flow by restricting vehicle access within a neighborhood. Motor vehicles are directed to major roads that surround the superblock, freeing up numerous internal streets for pedestrians and cyclists to use safely and comfortably. To evaluate the health effects of the implementation of these superblocks, a project was carried out, using both quantitative and qualitative methods and involving the participation of various areas and institutions. This project is called ‘Health in the streets’ (‘Salut als carrers’ in Catalan), developed during 2019–2021, aims to assess the potential environmental and health effects of the superblocks model from an equity perspective [8]. Its mains results are described in this paper.

Although they go by different names, these types of interventions that transform the city into greener, more walkable, and less traffic-heavy environments have been implemented in many cities around the world in recent years. There is strong evidence how traffic calming measures reduce the frequency and severity of collisions. The meta-analysis underataken by Elvik indicates that area-wide urban traffic calming measures reduce injury-related collisions by approximately 15% on average. Residential streets see the most significant decrease, with a reduction of about 25%, while main roads experience a smaller reduction of around 10% [9]. Grundy et al. reported 20 mph traffic speed zones are effective measures for reducing road injuries and deaths [10].

There is high evidence how urban and transport planning practices significantly influence greenhouse gas emissions, air pollution, green space availability, urban heat island effects and have a direct impact in health outcomes [11, 12].

Although there are many studies on the development of superblocks in Barcelona and other cities, there is little empirical evidence of their impact on health and well-being [13, 14]. The scoping review by Cash-Gibson et al. explores the health impacts of superblock interventions, which aim to redesign urban spaces to prioritize pedestrians, green spaces, and sustainable mobility. The study identifies potential benefits such as improved physical activity, mental health, and air quality. However, it highlights limited empirical evidence directly linking superblocks to health outcomes, emphasizing the need for further research to confirm these impacts and guide implementation effectively. They reported a surprising lack of empirical evidence on the effects of superblocks on health outcomes. In their review, they found only four papers with empirical data, three of which provided estimated effects by modelling in Barcelona and Los Angeles [14]. The study in Los Angeles adapted the Superblocks concept, showed significant reductions in hospital admissions and economic savings due to improved air quality and decreased traffic. The autors reported that the benefits were strongest when 5–10% of residential areas were transformed, but decreased as the threshold reached 30%. The study demonstrated how urban designs that reduce vehicle reliance can yield measurable health and economic benefit [15].

The objective of this study was to describe the effects on environmental, health and quality of life of the first three superblocks implemented in Barcelona, discuss the lessons learned, and provide recommendations for the future.

## Methods

**Fig. 1:**
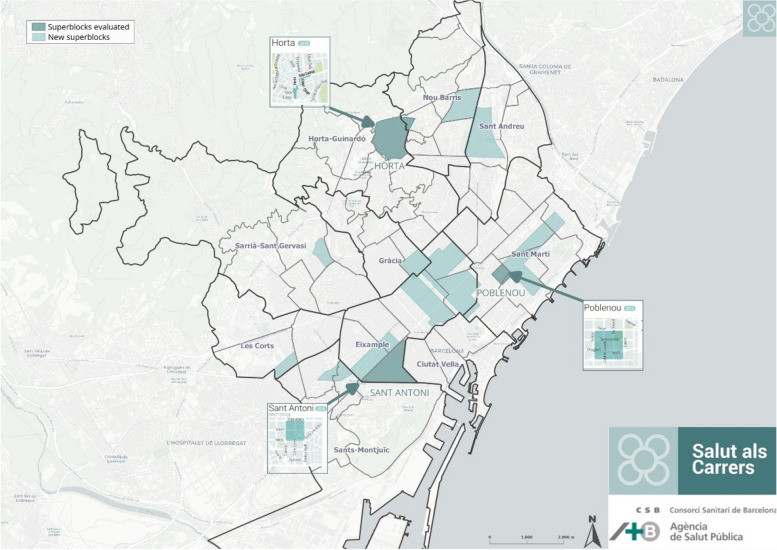
Planned and implemented Barcelona superblocks

When beginning the study, all the superblocks of the city involved different types of intervention at different stages of implementation. In Fig. 1, we can see a map of Barcelona with the different intervened areas and also the future superblocks.

**Fig. 2:**
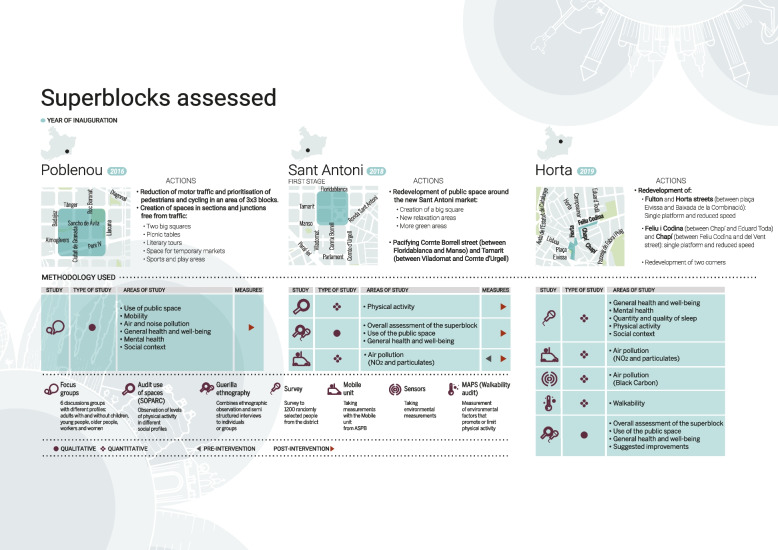
Description of the intervention in each superblock and methodology used to evaluate

The three superblocks assessed, as well as the interventions carried out, are described in Fig. 2 and Table 1. It comprised different methods, both quantitative and qualitative, which are listed in Table 2. The choice of one or other method depended mainly on feasibility and adequacy, as interventions were in different stages when we started the evaluation (Poblenou was finished, Sant Antoni was already under works, and Horta was in a planning phase) For example pre-post measures was only been available in Horta. But we tried to cover all the expected health outcomes based on our conceptual framework and implement complementary studies to have as much evidence as possible.

**Table 1:** Description of the interventions conducted in the 3 neighbourhoods (Palència et al., 2020)

**The Poblenou superblock**
The Poblenou superblock is a typical amalgamation of 3 × 3 blocks following the characteristics of the Eixample district in Barcelona. However, the superblock is located in an area that was originally industrial but is now home to many young families. The aim of the superblock is to calm the area of 3 × 3 blocks by reducing motorized traffic and prioritizing pedestrians and bicycles. The superblock consists of the creation of public spaces in some of the former roads and their intersections, including recreational areas (with picnic tables, literary tours, and space for future markets), play areas (with several areas devoted to children’s activities) and sports areas (with ping-pong tables, basketball hoops, and an athletics track). This superblock was completed in September 2016
**The Sant Antoni superblock**
Sant Antoni is situated in the center of Barcelona and has a high concentration of older residents. The first phase of the implementation of the superblock program in the Sant Antoni neighborhood (which is evaluated here) was completed in May 2018 with the urbanization of the surroundings of the Sant Antoni market. This phase included interventions on two streets (in total four sections of streets forming an intersection) and the creation of a new public square measuring 1800 m^2^ at their intersection. In total, 5000 m^2^ of public space is reserved for pedestrians, with sitting areas for new uses and a greater presence of greenery, including trees and bushes
**The Horta superblock**
This neighborhood has less traffic and noise than the other areas. Work on the superblock program in Horta started in October 2018 and mainly included the following interventions: 1) Traffic calming of the main entry street to the neighborhood, with the introduction of a level surface (no slope in the pavement) and a speed limit of 10 km/h to discourage the entry of vehicles to the neighborhood; 2) two streets with numerous public and private facilities, where there was previously almost no pavement, underwent various changes, including the creation of a level surface and removal of parking spaces; and 3) a tactical intervention (low-budget, temporary, and reversible) with removal of parking areas and the creation of sitting areas. The work was completed in December 2019

**Table 2:** Methods used in the evaluation of each superblock. Barcelona, 2018–2020

Superblock	Information source	Type of study	Areas studied	Measurement
**Horta**	Survey	Quantitative	General health and well-being, mental health, sleep quality, physical activity, social and neighborhood context	pre and post
	Sensors and NO_2_ passive samplers	Quantitative	Air pollution (NO_2_ and black carbon)	pre and post
	Walkability audit (MAPS)	Quantitative	Walkability	pre and post
	Ethnographic guerrilla	Qualitative	Global assessment of the superblock, use of public space, general health and well-being, proposed improvements	Post
**Sant Antoni**	Audit of the use of spaces (SOPARC)	Quantitative	Physical activity	post
	Ethnographic guerrilla	Qualitative	Global assessment of the superblock, use of public space, general health and well-being, proposed improvements	post
	Air quality mobile unit	Quantitative	Air pollution (NO_2_ and particulate matter)	pre and post
**Poblenou**	Focus groups	Qualitative	Use of public space, mobility, air pollution and acoustics, general health and well-being, mental health, social context	post

These methods included a health survey, environmental measures of air quality, the Microscale Audit of Urban Landscapes for Pedestrians (MAPS), qualitative studies and a System for Observing Play and Recreation in Communities (SOPARC). Details on the methodolgy used follow:

### Health survey

A pre-post intervention health survey (superblock of Horta) was conducted in a random sample of 1200 individuals before (May –June 2018) and after (September–October 2020) the intervention. The sample was extracted randomly from the municipal register of inhabitants with six age and gender quotas (200 in each quota). Respondents were individuals living in the neighborhood for at least six months. An interview was conducted in people’s homes by trained interviewers, and the same participants were followed in the two waves of the survey. The information analyzed was general self-perceived health (with the question that includes 5 possible answers: excellent, very good, good fair, poor) and well-being (measured trough de questionnaire EuroQol -EQ-5D-5L- with answers in a Likert scale), mental health (using the Goldberg questionnaire of 12 items with 4 possible answers each one), sleep quality (hours of sleep), physical activity (using the International Physical Activity Questionnaire), and the social and neighborhood context (7 questions with five-level likert scale responses based on the Community Life Survey and a battery of questions from different sources with 1 to 10 rating scales based on social and environmental aspects). Specific questions on the opinion regarding the intervention and effects of the superblock on the use of public spaces and mobility were prepared by the research team and asked only in the post-intervention survey [16–22].

The pre-questionnaire is included in Supplementary file 1 and the new questions included in the post-questionnaire are included in Supplementary file 2.

### Environmental measures of air quality

Pre- and post-measurements of critical air quality pollutants (NO_2_ and PM_10_ particles) were taken at a pedestrianized point of the Sant Antoni superblock (in the new public square). Measurements were taken with an equipped vehicle (mobile atmospheric control unit) which was set up during two time periods (pre-intervention, from 09/07/17 to 10/23/17, and post-intervention, from 10/01/18 to 10/16/18). The mobile atmospheric control unit is fitted with automatic analyzers to measure NO_2_ and PM_10_ particles according to the reference methods of Directive 2008/50/EC on ambient air quality and cleaner air for Europe.

In Horta, pre- and post-intervention NO_2_ and black carbon measurements were taken in May–June 2018 and September–October 2020, respectively, in 20 locations: 4 points in the intervention area; 9 points in areas close to the intervention (classified as affected controls which are close to the intervention area and could undergo a change in their traffic or air pollution levels) and 7 control points in areas sufficiently distant from the intervention (classified as controls). NO_2_ was determined using passive diffusion samplers (NO_2_ passive diffusion tubes, Gradko, UK) in duplicate (two tubes per point), continuously exposed for 4 full weeks at a height of 3 meters at each sampling point, and placed on supports such as poles or streetlights. Black carbon concentrations were measured using the microAeth AE51 instrument (microAeth, Model AE51 Magee Scientific, Berkeley, California, USA) at a recording frequency of 60 seconds and a flow rate of 150 mL/min. Measurements at each point were taken for 30 min on each sampling day, excluding peak hours Three passive diffusion samplers were installed in the nearby Governmental Fixed station, as well as a microAeth AE51 both for quality control purposes. Air quality measurements were compared and adjusted to reference stations from the city's fixed air quality network, reducing the effect of temporal variability due to weather conditions or other factors (i.e. COVID-19). No air quality data were collected for the Poblenou superblock.

### The Microscale Audit of Urban Landscapes for Pedestrians (MAPS)

The MAPS [23] collected audit data on microscale streetscape characteristics that influence the walkability of the built environment and physical activity of pedestrians (e.g. street design, transit stops, sidewalk qualities, street crossing amenities and features impacting aesthetics) in the Horta superblock. Before and after the implementation of the superblock, two independent auditors (one male and one female) completed the MAPS audit along four routes of the Horta street network.

### Qualitative studies

Two ethnographic guerrilla studies were carried out in Sant Antoni and Horta. This is a qualitative method that combines observation with short (3–15 min) semi-structured interviews with about 10 individuals or groups of people. Participants are not recruited beforehand but are approached spontaneously and are informed of the objective and characteristics of the study. Data are collected by means of multimedia with audio, video, and photographic recordings. Different profiles of people were explored: residents, users of the superblock, floating superblock users, merchants, and residents in the streets adjacent to the superblock. In addition, we attempted to ensure the maximum representation possible of different sociodemographic variables, such as age, gender, social class, and country of origin. The items explored were: overall opinion of the superblocks; strong points of the superblocks, i.e., positive contributions to daily life and quality of life in the neighbourhood; weak points or drawbacks; changes in patterns of use of the superblocks; perceived effects on health; and suggested improvements.

In addition, six focus groups were carried out to analyze the qualitative perception of the effects of the superblock among the people living, studying, or working in the Poblenou neighbourhood. The groups included 6–8 participants and consisted of 60–90 min of discussion with a moderator and an observer. The various groups were composed of parents of children living in or near the superblock, adolescents studying in the superblock, older people living in or near the superblock, people not living in or near the superblock but studying or working there, women living in or near the superblock and other adults not included in the other groups who lived in or near the superblock.

### Physical activity—Observing Play and Recreation in Communities (SOPARC)

To describe changes in the mobility and the use of spaces according to activity and age groups, we carried out an observational study on target areas of physical activity (SOPARC). SOPARC is a validated direct observation tool for assessing physical activity and associated contextual data characteristics in community setting [24, 25] which assessed patterns of space use, counting the number of people using the space and profiles and activities (Sant Antoni).

The study was approved by the Ethics Committee of reference (Parc Salut Mar Ethics Committee) with the reference number 2018/7979. More details of the methods are described in the project protocol [8]. It is worth mentioning that informed consent to participate was obtained from all of the participants in the study.

## Results

We present the results by outcome to provide all evidence by outcome together. And then, due to the differences by area, we present a summary of the results by each superblock. More detailed information can be found in Table 3.

**Table 3:** The main findings by superblock and outcome. Barcelona, 2018–2020

Superblock	Information source	Outcome	Main results
**Horta**	Survey	Mobility	90% of men and 85% of women visited the superblock at least once a week More than 60% of men and women believed that walking comfort had increased and about 75% of men and 70% of women that accessibility for strollers had improved Inner streets: More than 50% used them for walks or shopping, but only 6% used them for physical activity
	Survey	Health and well-being	55% of men and 45% of women believed that well-being in the intervention streets had increased 6% reported they used the intervention streets for physical activity
	Sensors and NO_2_ passive samplers	Air pollution	-Streets in the intervention area: NO_2_ from passive collectors decreased by between 17 and 27% Black carbon decreased considerably (by one of the two measured points) -Streets in the affected control and control area: No overall significant changes observed
	Walkability audit (MAPS)	Mobility	Improved microscale characteristics of the built environment influencing the walkability of the area and citizens’ physical activity, making this urban space more "activity-friendly"
	Ethnographic guerrilla	Mobility	-Entrance street to the neighborhood: Still too many vehicles (which exceed the speed limit and were stationary on the old sidewalks) Pedestrians needed dodge these stopped vehicles and walk on the road No pedestrian crossing Vehicles still circulated as if they had the priority Pedestrians felt less safe and more stressed than before, especially if accompanied by a child -Inner streets: There was a perceived decrease in vehicles and their speed There was a perceived increase in space for pedestrians and improved accessibility for people with reduced mobility The enhanced aesthetics favored conversation and spending time on the street There was a perception that the intervention encouraged conversation and spending time on the street
	Ethnographic guerrilla	Social support	- Inner streets: Enhanced aesthetics encouraged conversation and spending time on the street Walking in the Inner streets (superblocks) was enjoyed in a calmer and more pleasant way, with greater aesthetic appeal and opportunities for conversation and spending time on the street
	Ethnographic guerrilla	Health & well-being	In the inner streets walking was enjoyed in a calmer and more pleasant way, with a greater aesthetic appeal and opportunities for conversation and spending time on the street
**Sant Antoni**	Audit of the use of spaces (SOPARC)	Space use	There was a wider range of uses: people passing through, sitting, shopping, walking, playing, or exercising Greater use as a space to hang out Use of the superblock remained above 900 people per hour Only 2% of women and 6% of men engaged in vigorous physical activity in the superblock In general, the superblock was perceived as a safe space, with more open areas and better lighting
	Ethnographic guerrilla	Air pollution	There was a perception that reducing cars also reduced noise and pollution
	Ethnographic guerrilla	Space use	Few young people were observed and many older people The number of people using the superblock as a transit area to move from one place to another increased because it was perceived as a more pleasant route Despite the reduction in the number of cars, there was still too much traffic and motor vehicles exceeded the speed limit of 10 km/h
	Ethnographic guerrilla	Mobility	Families with children believed that the superblock allowed ease of mobility, but also that it led to a false sense of safety since there were still too many cars
	Ethnographic guerrilla	Social support	The superblock encouraged social engagement and there was less pollution and more space to be outdoors
	Ethnographic guerrilla	Health and well-being	The reduction in traffic noise increased the feeling of calm and relaxation The space was considered more full of life, more like a "neighborhood", a quiet, safe and comfortable place to spend time People highlighted the decrease in pollution and the greater space to be outdoors and sunbathe
	Air quality Mobile unit	Air pollution	NO_2_: 25% reduction in the superblock (a decrease of 14,6 µg/m^3^ in average) PM_10_: 17% reduction in the superblock (a decrease of 4,1 µg/m^3^ in average)
**Poblenou**	Focus groups	Air and noise pollution	Perception of a reduction in air pollution and especially in noise pollution Perception in some groups that pollution could have increased in the streets surrounding the superblock
	Focus groups	Space use	Groups making the greatest use of the superblock: • Families with children (especially women) in the children's play areas • Workers, for eating or at the end of the day Older adults did not use the superblock Young people believe that the superblock was not designed for them
	Focus groups	Mobility	Overall, there was less traffic in the superblock, but more traffic in the surrounding streets Mobility was improved for cyclists, roller skaters, and others Cyclists using the superblock stated that they felt safe despite the lack of cycle lanes More people chose to walk since private vehicles were not allowed in the superblock Perception of a lack of safety among pedestrians due to the presence of moving vehicles Older adults believed there were negative effects on mobility and access to certain places due to changes in public transport There was a perception that traffic could have moved to the streets surrounding the superblock There was tension in the coexistence between pedestrians and vehicles, and a lack of clear signage
	Focus groups	Social support	The superblock facilitated interaction among residents, fostering relationships and social networks Some women found the area to be deserted and perceived a certain lack of safety, while others perceived the opposite due to its being an open space
	Focus groups	Health and Well-being	There was a perception of a more relaxed atmosphere and a decrease in feelings of stress Overall, there seemed to be an improvement in mental health Diet improved in working people (due to more spaces for eating their own meals) and ease of walking

### Effects by each outcome

#### Air pollution

The results on air pollution differed according to the intervention area. The concentrations of initial pollutants at Sant Antoni were those of a heavy traffic situation in the city whereas in Horta the pollution levels fall within the ranges found at the city’s urban background stations. Air pollution decreased in the Sant Antoni superblock, with a 25% statically significant reduction in NO_2_ levels (a decrease of 14,6 µg/m^3^ in average, 95CI: −17.06; −12.14)) and a 17% decrease in PM_10_ particle levels (a decrease of 4,1 µg/m^3^, 95%CI: −5.06; −3.16) in the pedestrianized area (data from a mobile unit) where traffic was eliminated. The concentrations measured with the mobile unit located in the intersection intervened were compared with the fixed reference station in Eixample and average time differences before (2017) and after (2018) the intervention are shown.

No overall significant air pollution changes were observed in Horta, although small changes were detected in certain parts of the area where the intervention produced a reduction in traffic (17%−27% reduction in indicative measurements of NO_2_ and black carbon). For the points classified as affected control and control, no overall significant changes are observed. In terms of perception of air quality, half of the residents reported that air pollution had diminished in the intervention area, but only 10% of them believed that it had decreased in the surrounding streets, indicating that the improvement was limited to the intervention area of the Horta superblock (data from health survey).

#### Use of spaces

The use of spaces varied, with an overall increase in the use of the intervention area. In Sant Antoni, there was a larger presence of older people, but not of young people (data from SOPARC and ethnographic guerrilla studies). Families with children reported that the space allowed them to move around comfortably, but also generated stress, since it gave them a false sense of safety, as cars still passed by at a certain speed. In Poblenou (data from focus groups), families with children used the new children's play areas, while working people frequented it for lunch or at the end of the day. Other groups used it mainly as a place to pass through. Young people thought that the space was not designed for them, and older people did not use the superblock since it seemed an isolated space to them. Some women felt the area was deserted and perceived it to be unsafe, while others perceived the opposite, as it was an open space.

#### Physical activity

The results on physical activity and mobility varied (data from SOPARC). In Sant Antoni, most superblock users walked (92.9%), while only a few engaged in vigorous-intensity physical activity (3.1%), sat (3%) or stood (1%) [26]. Vigorous activities were more often performed by males (4.6%) than females (1.7%) [26].

In Horta, more than 50% used the superblock for walking or shopping, but only 6% used it for vigorous-intensity physical activity. More than 60% of people surveyed in the Horta neighborhood superblock felt more comfortable walking in the interior, narrower streets, and reported improved accessibility for strollers and people with reduced mobility (data from survey).

#### Health and quality of life

Overall, the qualitative survey identified benefits in emotional health, better rest, less noise and air pollution, and increased social engagement. The superblock created a more relaxed and less stressful environment, which encouraged walking, increased the feeling of tranquillity, and improved mental health. The space also encouraged interaction among residents, favoring social networks (data from survey and focus groups). In Horta, around 50% of people believed that noise had decreased and well-being had improved (data from survey).

### Specific effects of each superblock

#### Horta

The qualitative study (data from ethnographic guerrilla studies) showed mixed effects. In fact, two different parts of the neighbourhood were intervened: the main street of the neighbourhood that crosses it and the inner streets. Traffic calming in the inner streets succeeded as there were fewer cars, which travelled at a lower speed, pedestrians used the entire street to walk, and there was better access for people with reduced mobility. However, the intervention did not succeed on the main street of the neighbourhood, where there were still a large number of motor vehicles, traveling at high speeds and stationery on the former pavement, and pollution did not decrease. In addition, pedestrians had to walk in the road to avoid stationary cars. There were no pedestrian crossings and drivers behaved as though they had priority; consequently, the pedestrian experience was less safe and more stressful than before.

The pre-post superblock intervention MAPS audit study showed that the superblock improved microscale environmental attributes that influence pedestrians´ physical activity, indicating that the superblock model is a feasible approach to developing physical activity-friendly (more walkable) urban environments in city areas.

#### Sant Antoni

The superblock of Sant Antoni was rated highly (analyzed by data from the ethnographic guerrilla studies); in fact, it was the most highly valued superblock. The area encouraged socializing and walking in a quiet, safe and comfortable way. The area provided a feeling of greater peacefulness and better rest, and air quality was improved significantly. In terms of users, there was a large presence of adults and elderly people but few young people.

#### Poblenou

This superblock was extensively used by families with children and working people (data from focus groups). Young people thought that the space was not designed for them and elderly people did not use the superblock as they found it an isolated space There was a noticeable decrease in the perception of pollution and noise due to the reduced traffic but there was also concern that traffic may have moved to surrounding streets.

## Discussion

This study evaluated the recently implemented superblocks in Barcelona and found overall positive effects on a range of health indicators, although there was some variability in these effects, and they differed by superblock.

Residents and workers in all three of the evaluated superblocks reported a gain in perceived well-being, greater peacefulness and enhanced sleep quality, some reduction of noise and pollution, and an increase in social interaction. The built environment of the superblocks clearly influenced walkability, and a lower traffic volume led to improved air quality measures. In Horta, the same intervention seemed to work in one scenario of the intervention areas but not in another.

The intensity of the interventions carried out in the superblock areas improved environmental and health outcomes, because of traffic calming, leading to a reduction or elimination of traffic. However, the benefits were restricted to the pedestrianized streets or areas with a higher reduction in road traffic and were insufficient when traffic was not completely eliminated. In addition, future assessments are needed to evaluate the potential impact on air quality in the streets not intervened within the superblock area, which may act as traffic conduits and may create inequalities among residents [27].

Superblocks appeared to have a complex relationship with various aspects of city life. For air quality, the marked difference in results between Sant Antoni and Horta shows that the potential environmental benefits of these interventions may be considerably influenced by the intrinsic characteristics of each area. Firstly, the improvement in air quality due to traffic-calming interventions in areas with higher traffic and elevated pollutant concentrations is more effective than the improvement observed in urban background areas. In addition, the variability in the results shows that a more personalized approach, taking into account existing traffic patterns and road network configurations, could be crucial to maximize the benefits of these interventions. Secondly, when considering the use of public spaces, there are a number of critical questions about the inclusiveness of these interventions. The apparent divergence in perceptions and experiences among different demographic groups highlights a significant opportunity to recalibrate these interventions in a way that encompasses a broader demographic range, thus promoting inclusivity and reducing social inequalities [27, 28]. For physical activity, the data suggest a potentially rich opportunity to encourage more active lifestyles not only accessible but that also invite people to participate in a variety of physical activities. Finally, superblocks act as catalysts for a healthier and more connected urban environment, as shown by the health and quality of life surveys.

To address these challenges, however, a more integrated and holistic approach is needed, especially in terms of pedestrian safety and potential traffic reorganization. The lessons learned from Horta, Poblenou and Sant Antoni provide insight into how these interventions could be further refined to create urban spaces that are not only greener and quieter, but also encourage a sense of community and well-being among their residents [3, 13, 29].

The superblock program in Barcelona appears to be an important strategy to improve cities. It is in line with several global interventions that seek to reshape the urban environment in a more sustainable, healthy, and people-centered way [5, 29–32]. The interventions described focus on human well-being, the promotion of active mobility, and a sustainable approach, which is also found in other significant initiatives such as "Healthy Streets" [33, 34] in London and "Complete Streets" [35] in the United States. However, it is important to note the particular characteristics of superblocks that aim to revitalize and redefine the traditional urban spaces of Barcelona (blocks) by creating recreational and rest areas for local residents, blending tradition and modernity to respond to modern challenges. The superblocks attempt to change the traditional concept of the composition of the "street", which prioritizes cars by allocating most of the existing public space to them, over people.

### Strengths and limitations

“Salut als Carrers” project is highly innovative, employs different methodological approaches, and analyzes different outcomes to achieve this goal. The project has involved many scientific backgrounds to better achieve the project goals.

The main limitations to consider are the following: a) the analyzed superblocks affect small areas and, moreover, the expected changes in health outcomes are also small. Therefore, the expected results are also limited, making their measurements specially difficult; b) the timing of the implementations did not allow evaluation of major interventions (e.g., work in Sant Antoni started before the health survey took place, obliging us to change plans and conduct the survey in Horta instead, which was a less ambitious intervention); c) the absence of pre-intervention measures in some studies (such as SOPARC) prevented assessment of the magnitude of the change. It would be useful to have comparison groups in the evaluations but the complexity of all these evaluations did not allow us to add this aspect. However, the different methodologies used in different areas gave consistency as well introducing specificity and variability. d) the COVID-19 pandemic occurred during this period, which influenced the methodologies used and possibly some of the results observed (e.g., we could not analyze the health outcomes of the health survey since the COVID-19 pandemic occurred between the two waves of the survey and strongly influenced their results).

### Lessons learned

It is important to go beyond “Health in all policies” and progress to “Health for all policies” as argued by Greer [36]. This implies collaboration among sectors to create win–win solutions that design policies that provide co-benefits for multiple sectors. As we have shown, the superblock initiative is one of these policies. Moreover, including the health effects of a policy encourages acceptance of its implementation by the population. However, coordination with different sectors is still difficult, mainly due to the vertical organization of the public administration. It is important to acknowledge that working outside our silos improves the implementation and effects of policies. Therefore, cross-sectional work and good communication with the areas involved in the development of the intervention are essential.

Complex interventions are not easily evaluated. The processes and dynamics of their implementation affect the evaluation of health in natural experiments. Some aspects that should be taken into account are the following: a) Different moments of opportunity; the interventions implemented by a city council depend on multiple factors and therefore it is not always possible to know in advance all the details of the implementation, which would be ideal to plan a more robust evaluation; b) as previously mentioned, intersectoral coordination is not always easy, although it is essential in all steps of any intervention; c) the scale of innovative interventions is usually small at the beginning, and expands to other neighbourhoods of the city over the years, which increases the difficulty of evaluating the initial stages. However, despite these limitations, evaluating all the policies or interventions is essential to assess their potential effects and to identify potential issues for improvement.

## Conclusions

Although on a small scale, the present evaluation of the health effects of the superblocks supports expansion of the superblock model. These measures have the potential impact to the health of the population, but only if they are extensively implemented, as demonstrated elsewhere [32]. This is especially urgent in the context of the climate emergency, which should be a priority for action. Collectively, these interventions and similar ones around the world seem to complement each other, offering a strategy that could inspire and guide future global urban initiatives.

### Policy recommendations

The following are the main recommendations or improvements derived from the project carried out:

- Traffic calming actions within the superblock do not solve the problem of air pollution in the entire superblock territory and much less at the city level. More intensive and extensive traffic calming measures, as well as other actions, are needed to reduce traffic and emissions to finally improve air quality in a larger area. The selection of traffic-calming interventions should consider, among various prioritization factors, areas with higher levels of pollution, where they are most effective, and the most vulnerable populations (e.g. infants and elderly people).
- In several of the superblocks, there is a need for more street furniture and elements adapted for different age groups. It would be advisable to create areas that cater to different age groups to maximize the effectiveness of promoting physical activity. Different needs could be detected during the design of the intervention through increased participation of residents to avoid designs that exclude certain ages-genders or other population characteristics and exacerbate social inequalities.
- Signage and the creation of mechanisms need to be improved to further restrict private car access to the superblocks, reduce speed and emphasize pedestrian priority.
- The expansion of the space to spend time (especially in superblocks where little space has been created), as well as the increase in greenery have consistently been highlighted as important factors for enhancing the well-being of residents. The superblocks program provides an excellent opportunity to increase the physical activity and social interaction of the population by creating public, local, and outdoor urban spaces. In addition, increased greenery helps to mitigate the urban heat island effects and improve liveability and mental health [12].

## Supplementary Information

Supplementary Material 1.

Supplementary Material 2.

## Data Availability

The datasets used and/or analysed during the current study are available from the corresponding author on reasonable request.
