# Supplementary material for: Environmental and health effects of the Barcelona superblocks
Source: BMC Public Health. 2025 Feb 17;25:634. doi: 10.1186/s12889-025-21835-z (PMC11831821; doi:10.1186/s12889-025-21835-z)
Supplement: Supplementary file 1 — Supplementary Material 1. [file 12889_2025_21835_MOESM1_ESM.pdf]

CSB Consorci Sanitari de Barcelona

Agència de Salut Pública

Survey of health status of the population of the Superblocks of Barcelona (ESS) 2018

General questionnaire (population 18 years and over)

6 months or more viviento in the BARRIO SAN ANTONIO	If n☐ ☐	In case No ➤ *complete survey*
6 months or more living in the rest of the BCN district<br>(not include individuals residing in the San Antonio area)	If n☐ ☐	In case No ➤ *complete survey*

Questionnaire (registration) number	
Interviewer/a	
Date of interview	
Start time	
Time of completion (in *comments*)	
Holder or the person's name	

CONFIDENTIAL DATA

The information that we request in this questionnaire are to make an official statistic.

Administration and officials that use this information are obliged, by law, to statistical confidentiality, i.e., not to disclose it and not use it for any other purpose which is not the production of official statistics.

All citizens, organizations and institutions are required by law to provide the information requested, and this information must be complete and truthful.

(Articles 37, 38 and 39 of the law 23/1998 of 30 December)

A. Socio-demographic data

For all the people interviewed

1.1. Sex of the person interviewed

1 man
2 mujer

2 . 2. what is your date of birth complete?

Day	Month	Year

3. IDESCAT . With respect to people living in your home, which of the following fit more? (Read options)

1. a single person
2. two adults without children
3. more than two adults without children
4. an adult with one or more children
5. two adults with a child
6. two adults with two children
7. two adults with with more than two minors
8. more than two adults with one or more children

4.166. where he was born?

1. in the municipality of residence ➤ *go to question 6*
2. in another municipality of Catalonia**.** **Specify municipality** ______ ➤*go to question 6*
3. in another municipality in Spain, outside of Catalonia. **Specify province** ______ ➤*go to question 6*
4 overseas. **Specify country** ______

Only for people who have been born outside of Spain

5.167. in what year did Spain?

Year ______

For all the people interviewed

6. 117. How long does living at this address? *Choose the most appropriate time period*

1 life☐ ➤ *move to question 8*	years	months

7. 118. how long living in this neighborhood? *Choose the most appropriate time period*

years	months

8. 168. what is its maximum level of completed studies?

1 you can't read or write
2. incomplete primary: literate without having finished primary education
3. complete primary: primary LOGSE full or five courses of EGB

4. first stage of secondary education: graduate school, elementary school, school, or that full
5 high school studies: baccalaureate, BUP, baccalaureate new plan, PREU or COU

6 FP medium grade: industrial oficialia, FPI, middle grade training cycles
7 top grade FP: industrial expertise, formation, higher level training cycles

8. first cycle University studies: University diploma, architecture and engineering
9 second cycle University studies: degree, Bachelor's degree, architecture and engineering
10. University studies of doctorate, graduate, master, MIR or equivalent
11. Another possibility. **Specify.**

9 **. 189. What is your current job situation?**

1 works ➤ *go to question 10*
2 works, but you have a sick leave of more than 3 months ➤ *go to question 10*

3 standing to a subsidy/allowance
4 standing/a without subsidy/allowance

5 housework (housewife)

6 student

7 disabled/a or permanent disability

8 retired to age ➤ *go to pregunta10*

9 retired by the early ➤ *go to question 10*

10. Another situation. **Specify.**

10 . **190. although this person does not work remuneradamente today, would he had worked before?**

1 Yes
2. do not ➤ *go to question 12*

11 **. 191 . EPA survey active pob What is occupation or type of work that currently plays or played in your last job?** *(If the informant in doubt about the meaning of this question, some examples can be put as: bus driver, Builder's construction, Professor at the University...)*

➤ *go to question 13*

12 **. 191 . EPA survey active pob <u>With respect to the person who provides or provided most of the household, income</u> What is occupation or type of work that currently plays or played in your last job?** *(If the informant in doubt about the meaning of this question, some examples can be put as: bus driver, Builder's construction, Professor at the University...)*

B. State of health and quality of life

For all the people interviewed

Then we will make you a few questions concerning your health status

13.4. how would you which is your health in general? *Read*

1 excellent
2. very good
3. good
4 Regular
5. bad

14.5. During the past week how many hours has gone to sleep daily, average?

hours of sleep

15 . PISTBURG During the last month, during the last month how would rate, altogether, the quality of their hours of sleep?

1. very good
2 good
3. bad
4. very bad

16.8. Say which statements best described their health status in today. *Read by groups of 5 in 5 answers*

1. don't you have problems walking.
2 it has minor problems walking.
3 it has moderate problems walking.
4 he has problems walking.
5 you can't walk.

1. does not have problems for washing or dressing only / to
2 have minor problems for washing or dressing only / to
3 has moderate problems for washing or dressing only / to
4 has serious problems for washing or dressing only / to
5. cannot wash or dress only / to

1. don't have problems to do their daily activities (work, study, do the housework or leisure activities)
2 you have mild problems to make their daily activities
3 it has moderate problems to make their daily activities
4 it has serious problems to make their daily activities
5. you cannot do their daily activities

1. do not have pain or discomfort
2 you have pain or discomfort mild
3 you have pain or moderate discomfort
4 you have pain or discomfort strong
5 you have pain or discomfort end

1 isn't anxious / to or depressed / to
2 is slightly anxious / or depressed / to
3 is moderately anxious / or depressed / to
4 is very anxious / or depressed / to
5 is extremely anxious / or depressed / to

17. 9. We can state, on this scale it seems a thermometer, his opinion on his State of health generally in today? The 100 represents the best imaginable health state and 0 the worst imaginable health state.

Show thermometer and let the pencil or pointer so that the person being interviewed mark figure pollster/a:

His State of health today

Mark on the thermometer health today

Best imaginable health state

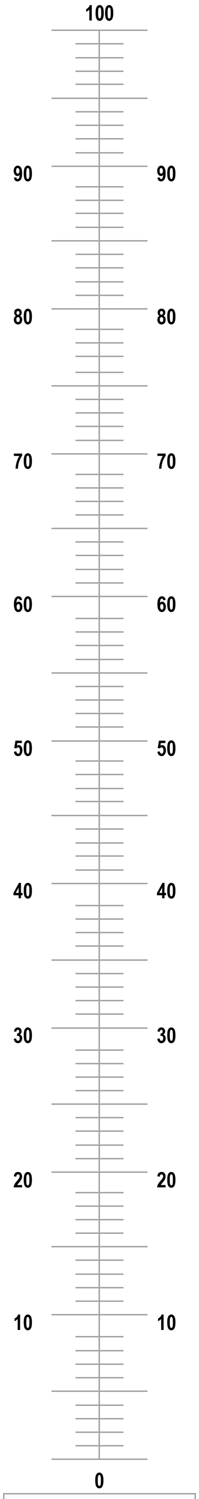

Worst imaginable health state

C. welfare

For all the people interviewed

18. (who5). Please indicate for each of the five statements which defines best how you have felt you during the last two weeks. Note that higher numbers mean greater well-being. *(Show card)*

During the last two weeks:	5. all the time	4. the largest part of the time	3. more than the half of the time	2. less than half of the time	1. of time in When	0. never
1. I have felt cheerful and in a good mood						
2. I felt calm and relaxed						
3. I have felt active and energetic						
4 I woke up me fresh and descandado						
5. my life has been full of things that interest me						

For all the people interviewed

19 . 27 . Then I will read you some phrases related to their social life and the emotional support that it receives from other people. Value from 1 to 5 If you have as much as I would like to (5) or less than what I would like to (1). *Show card*

	Less than what you want to				As much as you want
	1	2	3	4	5
1.tiene invitations to relax and hang out with other people					
2 you receive love and affection					
3 you have the possibility of talking to someone of their problems at work or at home					
4. you have the possibility of talking to someone of their personal and family problems					
5. you have the possibility of talking to someone of their economic problems					
6 you have people who care about what happens					
7 useful tips you receive when you miss something important in life					
8 get help when you are sick in bed					

D. mental health

For all the people interviewed

20. 26. The following questions relate to problems that has been to the last 30 days. Please choose the answer that most approaches what he feels or has felt recently

1. what has been able to concentrate well in what he was doing?	1	☐	Y es	better than usually
	2	☐	Y es No	as usually
	3	☐	No	less than usually
	4	☐	No	much less than commonly
2. what concerns have you losing too much sleep?	1	☐	No	Not at all
	2	☐	No Yes	no more than usually
	3	☐	Yes	a little more than usually
	4	☐	Yes	much more than usually
3. have you felt that it plays a useful role in life?	1	☐	Y es	more useful that usually
	2	☐	Y es No	as usually
	3	☐	No	less useful that usually
	4	☐	No	much less useful that usually
4. What have you felt able to take decisions?	1	☐	Y es	more than usually
	2	☐	Y es No	as usually
	3	☐	No	less than usually
	4	☐	No	much less than commonly
5. what we have constantly seen loaded to and live?	1	☐	No	Not at all
	2	☐	No Yes	no more than usually
	3	☐	Yes	a little more than usually
	4	☐	Yes	much more than usually
6 have you ever had the feeling that can not overcome their difficulties?	1	☐	No	Not at all
	2	☐	No Yes	no more than usually
	3	☐	Yes	a little more than usually
	4	☐	Yes	much more than usually
7 have you been able to enjoy daily activities?	1	☐	Y es	more than usually
	2	☐	Y es No	as usually
	3	☐	No	less than usually
	4	☐	No	much less than commonly
8. what has it been able to cope adequately, their problems?	1	☐	Y es	more capable that usually
	2	☐	Y es No	as usually
	3	☐	No	less capable that usually
	4	☐	No	much less capable
9. How have felt little happy and depressed / to?	1	☐	No	Not at all
	2	☐	No Yes	no more than usually
	3	☐	Yes	a little more than usually
	4	☐	Yes	much more than usually
10. How has lost confidence in itself / to?	1	☐	No	Not at all
	2	☐	No Yes	no more than usually
	3	☐	Yes	a little more than usually
	4	☐	Yes	much more than usually
11. have you ever thought that it is a person that is useless?	1	☐	No	Not at all
	2	☐	No Yes	no more than usually
	3	☐	Yes	a little more than usually
	4	☐	Yes	much more than usually
12 do you feel reasonably happy, considering all the circumstances?	1	☐	Y es	more than usually
	2	☐	Y es No	about the same as usually
	3	☐	No	less than usually

4	☐	No	much less than commonly

E lifestyles: physical activity

For all persons under the age of 65. If you are older than 65 years ➤ go to question 28

Sedentary behavior

Sedentary behaviors: Sedentary behaviors are activities that usually involve sitting down and not move, such as watching TV, etc.

21. Marshall-1. Please indicate how many hours a day is sitting normally during a weekday and a non-working day for each of the different activities:

	Weekday		Non-working day	
	1 hours	2 minutes	3 hours	4 minutes
1. in displacement (public transportation or car)				
2 watching TV (also to include DVDs, Tablet, Xbox, Playstation...)				
3. using your computer at home				
4. free time: reading books, listening to music, sewing, crafts, sitting in the Park, etc. (there is no watching TV or using the computer at home)				

Physical activity

22 . IPAQ SHORT 88 . Dd uring the last 7 days, you walked many days at least 10 continuous minutes in your free time?

days during the last 7 days

If it has not walked any day at your leisure ➤ go to question 24

23 . IPAQ SHORT 89 . Few minutes approximately, or average, has walked every day that made it?

minutes / day

24 . IPAQ SHORT 90 . Think only about those physical activities that made at least 10 continuous minutes. During the past 7 days, how many days did you VIGOROUS physical activities such as aerobics, running, pedaling fast bike, or swim faster in his time?

days during the last 7 days

If not already done any strenuous activity ➤ go to question 26

25. IPAQ SHORT 91. Few minutes approximately, or average, has made a vigorous activity every day that made it?

minutes / day

26 . IPAQ SHORT 92 . Again, think only about those physical activities that made at least 10 continuous minutes. During the last 7 days, how many days did you moderate physical activities such as pedal bicycle at a regular distance, swimming at a regular distance, play tennis doubles, in his spare time?

days during the last 7 days

If not already done any moderate activity ➤ go to question 35

27 . IPAQ SHORT 93 . Few minutes approximately, or as average has been a moderate daily which has done?

minutes / day

For all persons over the age of 65. If it is less than 65 ➤ go to question 35

Physical activity

Now I'd like to ask you about certain types of activities that it has made during the last month. I will ask about the amount of strenuous activity, slow walks, time sitting, standing (a) and some other things, which makes regularly.

28. Yale part two-1 have approximately, how many times in the month participated in vigorous activities, which lasted more than 10 minutes caused significant increases in your breathing, heart rate, and fatigue in the legs, and led him to sweat ?

1. no time *if it has not participated in any vigorous activity in the last month➤ go to question 30*
2. from 1 to 3 times per month
3. from 1 to 2 times per week
4. from 3 to 4 times a week
5. 5 or more times a week

6 he preferred not to answer

7. don't know

29. Yale part two-2 do approximately how long engaged you every time these vigorous activities?

1. do not apply
2. from 10 to 30 minutes
3. from 31 to 60 minutes
4. 60 or more minutes

5 preferred not to answer

6. don't know

30 . Yale part two-3 Think of the times that it has walked in the last month. Approximately, how many times in the month walked at least 10 minutes or more without stopping, while activity was strenuous enough to cause significant increases in your breathing, heart rate, or tiredness in the legs, or that take you to? do sweat?

1. no time *if done these walks in the last month➤ go to question 32*
2. from 1 to 3 times per month
3. from 1 to 2 times per week
4. from 3 to 4 times a week
5. 5 or more times a week

6 he preferred not to answer

7. don't know

31 . Yale part two-4 When he was walking this way, do for few minutes it did?

1. do not apply
2. from 10 to 30 minutes
3. from 31 to 60 minutes
4. 60 or more minutes

5 preferred not to answer

6. don't know

32 . Yale part two-5 approximately how many hours a day spend doing things that require you to stand and move? Please only report cases in which they are really moving.

1. no

2. less than an hour a day

3. from 1 to 3 hours a day

4. from 3 to 5 hours a day

5. from 5 to 7 hours a day

6. more than 7 hours a day

7 preferred not to answer

8 don't know

33 . Yale part two-6 consider the amount of time that is spent standing or moving over their feet during an ordinary day in the last month. Approximately how many hours a day is stopped (a)?

1. no

2. less than an hour a day

3. from 1 to 3 hours a day

4. from 3 to 5 hours a day

5. from 5 to 7 hours a day

6. more than 7 hours a day

7 preferred not to answer

8 don't know

34 . Yale 7. Approximately how many hours a day spent sitting (a) in an ordinary day in the past month? *Read*

1. no

2. less than three hours a day

3 3-6 hours a day

4 6-8 hours a day

5. more than 8 hours a day

6 he preferred not to answer

7. don't know

F mobility

For all the people interviewed

Them following questions made reference to the movements made on weekdays (from Monday to Friday), for instance yesterday (or Friday) If yesterday was Sunday.

DISPLACEMENT definition: refers to displacement as a full path that is made from a place (source) to another (destination) for any reason and that can make walking or using one or more modes of transport.

35. 125. EMEF 2017P2a. Yesterday (or the last working day) left you home?

1 Yes *If left home* ➤ *move to question 37*

2. DO NOT

36. EMEF 2017P2b. Why did not you House?

1. disease, disability

2 I was on vacation

3 he didn't exit

4. other. Specify

37. 126. How much time invested in total in each type of transportation? (adding all movements in a day by every means of transport) *(For example, travel on foot: 7 minutes to get to the subway + 8 minutes from the metro to work + 17 minutes up to the school to pick up children + 10 minutes from metro home = 42 minutes)*

	125. It has moved in:		126. total minutes on the last working day
	1 Yes	2.	
1. walk			
2. by bus			
3. by subway or tram			
4. by train			
5. on a motorcycle			
6. in moped			
7. bicycling			
8. by car			
9. in another medium. **Specify.**			

For all the people interviewed

*Them following questions made reference to the movements made in days **not working** (Saturday or Sunday), for instance yesterday (or Sunday) If yesterday was Tuesday.*

38. 125. EMEF 2017P2a. Yesterday (or the last non-working day) left you home?

1 Yes *If left home* ➤ *go to question 41*

2. DO NOT

39. EMEF 2017P2b. Why did not you House?

1. disease, disability

2 I was on vacation

3.He didn't want to leave

4. other. Specify

40. 126. How much time invested in total in each type of transportation? (adding all movements in a day by every means of transport) *(For example, travel on foot: 7 minutes to get to the subway + 8 minutes from the metro to work + 17 minutes up to the school to pick up children + 10 minutes from metro home = 42 minutes)*

	125. moved at		126. total minutes made in non-working days
	1 Yes	2.	
1. walk			
2. by bus			
3. by subway or tram			
4. by train			
5. on a motorcycle			
6. in moped			
7. bicycling			
8. by car			
9. in another medium. **Specify.**			

G. environmental context

For all the people interviewed

41. Kantar. Can I tell to what extent is agreed or disagreed with the following aspects of your neighborhood?

	1. very of agreement	2. of the agreement	3 agreement nor in disagreement or	4. at odds	5. very much in disagreemen t
1 I like living in my neighborhood					
2. my neighborhood is improving					
3 in my neighborhood, people from different countries of origin and ethnic groups get along well					
4 you can rely on the people in the neighborhood					
5 you can walk safely at night in my neighborhood					
6 I can influence decisions that affect my neighborhood					

42 . NEWS I Neighborhood satisfaction. Be then provided information about their neighborhood with which can be satisfied or not. Please indicate your level of satisfaction with each situation on the following scale: where (1) is equivalent to Very dissatisfied and (10) to Very satisfied.

Are satisfied with the following?

	Very dissatisfied	1	2	3	4	5	6	7	8	9	10	Very satisfied
How easy and pleasant that is walking in your neighborhood												
How easy and pleasant that is cycling in your neighborhood												
The amount and speed of traffic in your neighborhood												
The noise of traffic in my neighborhood												
The noise of the people in my neighborhood												
The amount of green spaces in my neighborhood (trees, plants, grass, flower pots, etc...)												
The offer of shops of fruit and vegetables in your neighborhood												
The price of fruit and vegetables in their neighborhood stores												
The amount of spaces of social interaction in my neighborhood *(e.g. squares, parks, etc.)*												
The number of tourists in my neighborhood												
Your neighborhood as a good place to raise children												

I coexistence, characteristics of the housing and economic situation

For all the people interviewed

Now we will make you a few questions about your home and the equipment which has

43. 149. what is ownership of your home? *Read*

1. of property (fully paid)
2. of property (paying mortgage)
3. in normal rental (market price)
4. for social rent
5. in re-renting of part of a floor

6 granted by social services or NGOs (Caritas, etc.)
7 given by family or friends
8. Another situation. **Specify.**

44. 149. is there some element that makes it difficult for housing access to street and vice versa? (E.g. stairs, steps, drops, etc...)

1 Yes
2. not *if there is any element that hinders access to housing* ➤ *go to question 46*

45. ONCE available in your building:

	1 Yes	2. do not
1 ramp		
2 platform lift		
3 lift		
4. other **specify.**		

46. hopes to change housing or is forced / to a changed housing during the next 6 months? Read

1. Yes - see me forced to change my housing
2. yes - I have plans to change housing on a voluntary basis ➤ *move to question 66*
3. not - I do not change my housing plans or I will be forced / to make it ➤ *move to question 66*
4. Another situation. **Specify.**

47. What is the main reason why will be oblidago/a change of housing? Read

1 I have to leave the House, because the owner has decided to terminate or not renew the lease.
2 I have to leave the House, because the owner has decided this in the absence of a rental contract.
3 I have to leave the House because of a warrant of eviction
4 I have to leave the House because of financial difficulties (e.g. not to be able to continue paying the rent or mortgage)
6. Another situation. **Specify.**

48. 162. considering the net monthly income of all members of their household, how to used to make ends meet?

1. with much difficulty
2. with difficulty
3. with some difficulty
4. with relative ease
5. with ease
6. with ease

Authorization consent

Only for people 18 years and older

49 . To end, we would like to know if you would agree to participate in the project's evaluation of the superblock.

I understand that the aim of the project is the assessment of the effects of the program on the environment and people's health. Therefore, I accept the release of data that may arise from my participation, which will allow the subsequent dissemination thereof, respecting the anonymity and within strict confidentiality.

1. Yes, would agree ➤ write down your name, the date and the f*irma authorization*
2. do not ➤ *passes to the 69-question*

Nombre:_

Date

Firma:_

50 . 200. If in the future we would like to return to contact you from the public health agency of Barcelona to ask her some questions **sheld its savalanche, would could return to? contact you**?

1. Yes, you don't mind to become to get in touch with you about this issue
2. , prefer that no is back to contact you about this issue

Thanks a lot.

The questions in the questionnaire **and**survey of the State of **S**avalanche of the population of the **S**upermanzanas (**ESS**) of Barcelona are already over.

Don't forget to ask for the phone number to facilitate the recontacto

1 facilitates phone number ➤ *write down it on the sample sheet*
2 refuses to give the number
3. do not have phone
4. do not know the number

Do not forget to request email to facilitate the recontacto

1 facilitates email ➤ *write down it on the sample sheet*
2 refuses to provide the valid e-mail address 3. It has no email
4. do not know your email address

Observation sheet *(to be filled in by the interviewer)*

A time of completion of the interview

Time

B. total duration of the interview in minutes

minutes

C. licensee or substitutes

1 owner☐	Substitute/a number	

D. the person being interviewed, have had any problems of comprehension or expression in the language in which the interview was conducted?

	1. any difficulty	2. any difficulty	3. many difficulties
Catalan			
Spanish			

E value 1 (very little) to 10 (very much) the attitude of the respondent as regards...

	1	2	3	4	5	6	7	8	9	10
1. the facility to provide answers										
2. the degree of sincerity										

F comments, or remarks
