# Supplementary material for: Environmental and health effects of the Barcelona superblocks
Source: BMC Public Health. 2025 Feb 17;25:634. doi: 10.1186/s12889-025-21835-z (PMC11831821; doi:10.1186/s12889-025-21835-z)
Supplement: Supplementary file 2 — Supplementary Material 2. [file 12889_2025_21835_MOESM2_ESM.pdf]

CSB Consorci Sanitari de Barcelona

Agència de Salut Pública

Survey of health status of the population of the Superblocks of Barcelona (ESS) POST

General questionnaire (population 18 years and over)

Questionnaire (registration) number	
Interviewer/a	
Date of interview	
Start time	
Time of completion (in *comments*)	
Holder or the person's name	

CONFIDENTIAL DATA

The information that we request in this questionnaire are to make an official statistic.

Administration and officials that use this information are obliged, by law, to statistical confidentiality, i.e., not to disclose it and not use it for any other purpose which is not the production of official statistics.

All citizens, organizations and institutions are required by law to provide the information requested, and this information must be complete and truthful.

(Articles 37, 38 and 39 of the law 23/1998 of 30 December)

J. THE SUPERBLOCK

Next, I will ask you about the pedestrianization and traffic calming works that took place at the Fulton, Horta, Chapí and Feliu Codina streets, that started in October 2018 and finished at the end of 2019.

101. Do you know the works that have taken place in Fulton, Horta, Chapí and Feliu Codina streets?

Yes
No ➤ *go to question 103*
(DO NOT READ) Prefer not to answer ➤ *go to question 103*
(DO NOT READ) Don't know ➤ *go to question 103*

102. In general how do you value the results of these works? (Read options)

Very negatively
Negatively
Netiher negative nor postively
Positively
Very positively
(DO NOT READ) Prefer not to answer
(DO NOT READ) Don't know

103. How many times a week do you pass by these streets? (Read options if necessary)

No times a week ➤ *go to question 105*
1 or 2 times a week
3 or 4 times a week
5 or 6 times a week
Every day of the week
(DO NOT READ) Prefer not to answer ➤ *go to question 105*
(DO NOT READ) Don't know ➤ *go to question 105*

104. What kind of activities you do there? (Spontaneous, Multianswer)

Walking
Buying
Passing by
Doing sport
Cultural or social activities
Others (write down)
Prefer not to answer
Don't know

105. In comparison with two years ago (before October 2018) how often do you walk or pass by these streets? (Read options)

Much less than before
Less than before
Same as before
More than before
Much more than before
(DO NOT READ) Prefer not to answer
(DO NOT READ) Don't know

106. Let's talk about the consequences of these works. You think that the following things have decreased, have not changed, have increased?

106.1. The level of comfort when walking by the intervened streets?
106.2. The accessibility for strollers, shopping carts and people with reduced mobility in the intervened streets?
106.3. The number of parking places in the intervened streets?
106.4. The noise in the intervened streets?
106.5. The noise in the surrounding streets?
106.6. The traffic in the intervened streets?
106.7. The noise in the intervened streets?
106.8. The air pollution in the intervened streets?
106.9. The air pollution in the surrounding streets?
106.10. El wellbeing of the neighbours in the interviened streets?
106.11. El wellbeing of the neighbours in the surrounding streets?

Has decreased
Has not changed
Has increased
(DO NOT READ) Prefer not to answer
(DO NOT READ) Don't know

107. Do you want to make another comment on the effects of these workds? (Spontaneous, write literaly)

K. COVID-19

Finally, I would like to ask you about COVID-19 and some consequences that may have had on your daily life.

201. Have you presented symptomatology compatible with COVID-19? (Fever, dry cough, tiredness, nasal congestion, headache, conjunctivitis, sore throat, diarrhea, loss of taste or smell, a rash on skin, or discolouration of fingers or toes).

Yes
No
(DO NOT READ) Prefer not to answer
(DO NOT READ) Don't know

202. Have you had a test to detect if you were infected by COVID-19ID-19?

Yes
No ➤ *go to question 204*
(DO NOT READ) Prefer not to answer ➤ *go to question 204*
(DO NOT READ) Don't know ➤ *go to question 204*

203. What was the result of the test?

Positive (you had or had had COVID-19)
Negative (you did not have or had not had COVID-19)
(DO NOT READ) Prefer not to answer
(DO NOT READ) Don't know

204. (Except if P9=5,7,8,9 in 2018 and P9=5,7,8,9 now) In general, during most part of the different phases of the COVID-19 lockdown, did you work in hospitals, clinics, socio-sanitary centres, day care centres or geriatric centres?

Yes
No
(DO NOT READ) Prefer not to answer
(DO NOT READ) Don't know

205. (Except if P9=5,7,8,9 in 2018 and P9=5,7,8,9 now) In general, during most part of the different phases of the COVID-19 lockdown, did you work in other essencial services such as supermarkets, transports, logistics, etc.?

Yes
No
(DO NOT READ) Prefer not to answer
(DO NOT READ) Don't know

206. (Except if P9=5,7,8,9 in 2018 and P9=5,7,8,9 now) In general, during most part of the different phases of the COVID-19 lockdown, did you do telecommuting?

Yes
No ➤ *go to question 207*
(DO NOT READ) Prefer not to answer ➤ *go to question 207*
(DO NOT READ) Don't know ➤ *go to question 207*

206.1. How many hous a day approximately?

|__|__| hours
(DO NOT READ) Prefer not to answer
(DO NOT READ) Don't know

207. In general, during most part of the different phases of the COVID-19 lockdown, did you take care of children or dependent people?

Yes
No ➤ *go to question 208*
(DO NOT READ) Prefer not to answer ➤ *go to question 208*
(DO NOT READ) Don't know ➤ *go to question 208*

207.1. How many hous a day approximately?

|__|__| hours
(DO NOT READ) Prefer not to answer
(DO NOT READ) Don't know

208. During most part of the different phases of the COVID-19 lockdown, how many people lived in your home (including you)? Concretament...

208.1. How many underage (0-17 years old)?
208.2. How many adults 18 to 64 years old?
208.3. How many people aged 65 or older?

|__|__|
(DO NOT READ) Prefer not to answer
(DO NOT READ) Don't know

209. Of your home, could you tell me...

209.1. Its surface in square metres approximately?

|__|__|__| m^2
(DO NOT READ) Prefer not to answer
(DO NOT READ) Don't know

209.2. Number or rooms except dining room, bathroom or kitchen?

|__|__| rooms
(DO NOT READ) Prefer not to answer
(DO NOT READ) Don't know

209.3. Does it have a balcony?

Yes
No
(DO NOT READ) Prefer not to answer
(DO NOT READ) Don't know

209.4. Does it have windows facing the street?

Yes
No
(DO NOT READ) Prefer not to answer
(DO NOT READ) Don't know

209.5. Does it have a terrace, with exclusive or shared access?

Yes
No
(DO NOT READ) Prefer not to answer
(DO NOT READ) Don't know

210. (Except if P9=5,7,8,9 in 2018 and P9=5,7,8,9 now) Because of COVID-19 you laboral situation has worsen, has not changed, has improved?

Has worsen
Has not changed
Has improved
(DO NOT READ) Prefer not to answer
(DO NOT READ) Don't know

211. (Except if P9=5,7,8,9 in 2018 and P9=5,7,8,9 now) Because of COVID-19 have you been part of a temporary labour force adjustment plan (ERTO)?

Yes
No
(DO NOT READ) Prefer not to answer
(DO NOT READ) Don't know

212. (Except if P9=5,7,8,9 in 2018 and P9=5,7,8,9 now) Because of COVID-19 have you lost your job?

Yes
No
(DO NOT READ) Prefer not to answer
(DO NOT READ) Don't know

213. How many stress do each of these aspects of your life provokes to you? (Read answer options)

213.1 Worry about you being infected by coronavirus.
213.2 Worry about one of your beloved ones being infected by coronavirus.
213.3 Death of a beloved person by coronavirus.
213.4 Lost of work or income beacause of coronavirus.
213.5 Negative or alarming messages about coronavirus in the media
213.6 Your health
213.7 The health of your beloved ones
213.8 Your sentimental life
213.9 The relationship with your family
213.10 Other problems your beloved ones may have
213.11 Your finantial situation
213.12 (Only if P9=1,2,10) Problems to deal with your workmates
213.13 Your life in general

Very intense
Intense
Moderate
Mild
Not at all
(DO NOT READ) Prefer not to answer
(DO NOT READ) Don't know

We have finishes.

TELF. Could you confirm your telephone number in case we have any doubt?___________________
